# Community-serving research addressing climate change impacts on vector-borne diseases

**DOI:** 10.1016/S2542-5196(24)00049-4

**Published:** 2024-05

**Authors:** Luis Fernando Chaves, Mariel D Friberg, Mercedes Pascual, Jose E Calzada, Shirley Luckhart, Luke R Bergmann

**Affiliations:** Department of Environmental and Occupational Health, School of Public Health and Department of Geography, Indiana University, Bloomington, IN, USA; Instituto Conmemorativo Gorgas de Estudios de la Salud, Ciudad de Panamá, Panama; Earth System Science Interdisciplinary Center, University of Maryland, College Park, MD, USA; Earth Science Division, NASA Goddard Space Flight Center, Greenbelt, MD, USA; Department of Biology and Department of Environmental Studies, New York University, New York, NY, USA; Instituto Conmemorativo Gorgas de Estudios de la Salud, Ciudad de Panamá, Panama; Department of Entomology, Plant Pathology and Nematology and Department of Biological Sciences, University of Idaho, Moscow, ID, USA; Department of Geography, University of British Columbia, Vancouver, BC, Canada

## Abstract

The impacts of climate change on vector-borne diseases are uneven across human populations. This pattern reflects the effect of changing environments on the biology of transmission, which is also modulated by social and other inequities. These disparities are also linked to research outcomes that could be translated into tools for transmission reduction, but are not necessarily actionable in the communities where transmission occurs. The transmission of vector-borne diseases could be averted by developing research that is both hypothesis-driven and community-serving for populations affected by climate change, where local communities interact as equal partners with scientists, developing and implementing research projects with the aim of improving community health. In this Personal View, we share five principles that have guided our research practice to serve the needs of communities affected by vector-borne diseases.

## Hypothesis-driven and community-serving research

Climate change has become a major concern for the long-term survival of life on Earth.^1^ Climate action, taking urgent action to combat climate change and its impacts, is one of the 17 Sustainable Development Goals set by the UN, which also include ending poverty and ensuring health and wellbeing for everyone.^2^ At the intersection of poverty, absence of access to health care, and climate change, vector-borne diseases have always been present. An example of this intersection is malaria, globally the most important vector-borne disease in terms of morbidity and mortality, causing over 600 000 deaths from 247 million cases in 2021, and mostly occurring in sub-Saharan Africa, a region where climate change converges with exacerbated poverty and weak health-care systems.^3^

In addition to malaria, clear linkages between climate and changes to disease prevalence have been documented for many neglected tropical diseases, such as leishmaniasis,^4,5^ and other diseases resulting from pathogens transmitted by bloodsucking insects. Many other non-insect-related neglected tropical diseases are sensitive to weather changes, including snakebites.^6^ Moreover, heatwaves and other extreme weather events also have direct impacts on overall human morbidity and mortality, and health systems as a whole.^7^ Under these circumstances, the need to perform research that improves our ability to cope with the impacts of climate change health has been recognised as a priority.^8^

**Figure 1:**
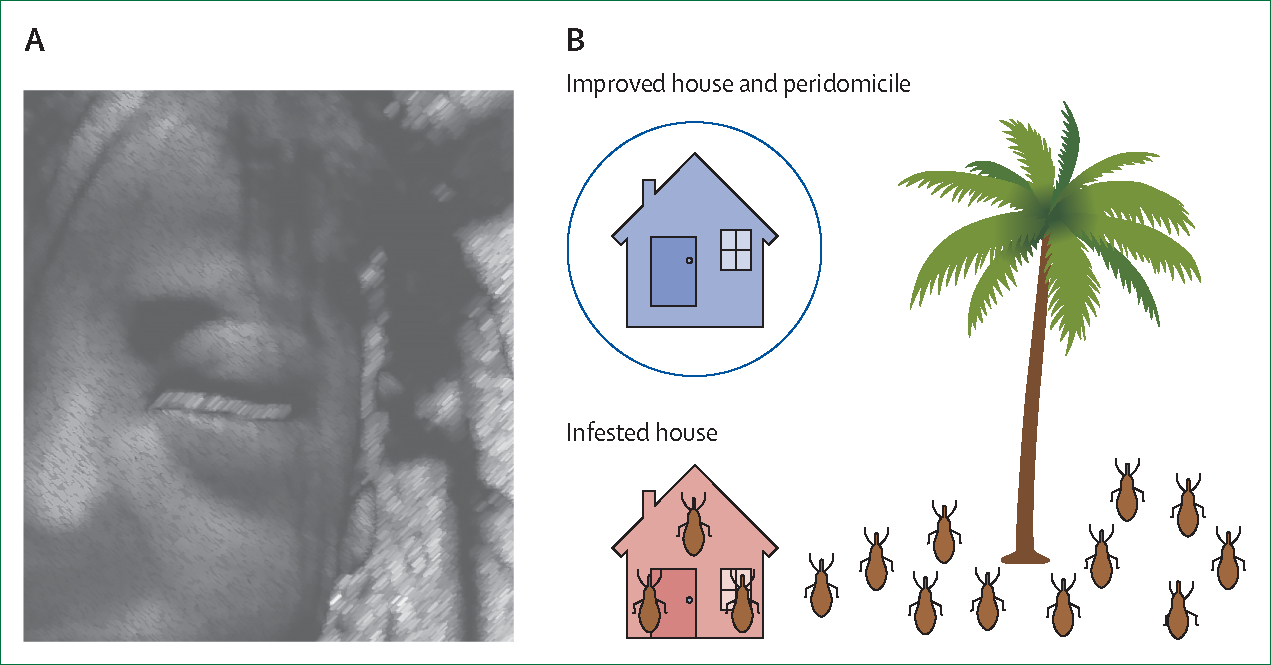
Clinical signs recognised by rural populations throughout Latin America as indicators of *Trypanosoma cruzi* transmission and infection risk (A) The Romaña sign, a unilateral bipalpebral oedema during the acute phase of *T cruzi* infection via the conjunctiva. This illustration is based on a picture originally published by Beucler and colleagues.^22^ (B) Parasite transmission to humans is normally mediated by kissing bugs. *Rhodnius prolixus*, one of the most efficient *T cruzi* vectors, normally lives in palms, but it easily colonises houses with mud walls and thatched roofs.^14^ In general, the most successful interventions to control Chagas disease have been based on environmental management to prevent kissing bug infestations.^19^ This figure was created with BioRender.com.

In this Personal View, we discuss the potential for research that is both hypothesis-driven and community-serving for populations affected by climate change. We define community-serving biomedical research as driven by the health needs of a local population and pursued with methods that actively involve local populations, through the coproduction of research-based knowledge that is useful in decision-making contexts and emerges from meaningful interaction between producers and users of research.^9^ The results of community-serving research are expected to improve the health and wellbeing of the local population by at least increasing the local populations’ understanding of health problems in their environment. Among vector-borne diseases, Chagas disease illustrates how hypothesis-driven and community-serving research can be successfully combined (panel 1; figure 1).

Community-serving research is common in fields outside the biomedical sciences, and we acknowledge that some of the ideas we propose here echo those developed in participatory cartography as pioneered by Gwendolyn Warren and the late William Bunge.^23–25^ Through urban geographical expeditions, most prominently in Detroit in the late 1960s, Bunge, a geographer, and Warren, a community activist, documented a series of inequities in the wellbeing of racialised populations, as illustrated by higher mortality risks and less leisure space for Black children compared with other children in Detroit. All of the knowledge gained through scientific inquiry was pursued with, by, and for the local Black population. Research questions were crafted by community members who best understood the problems that needed action able knowledge. Through collaborations between the community and scholars like Bunge, cocreated actionable knowledge was used to solve many urgent problems in Detroit.^26^ Seeking to adapt the methods of Bunge, Warren, and others,^9,27^ we present the following five practices to grow and strengthen community-serving research on the impacts of climate change on vector-borne diseases: (1) understand the local context of vector-borne disease transmission; (2) render knowledge actionable; (3) ask what else beyond climate change is affecting vector populations and transmission; (4) ask how the scope of research-based knowledge can be broadened; and (5) support communities to keep (or take back) what is theirs.

These practices can facilitate knowledge translation to reduce transmission and improve population health and wellbeing. From the perspective of community-serving research in vector-borne diseases, we also want to emphasise that these steps could be generalised to other health problems. Initially, the perceived impacts of vector-borne diseases might emerge from community members, as illustrated by the inference that Romaña’s sign and kissing bug housing infestations are warning signals of Chagas disease (figure 1). However, perceived impacts can also derive from statistical data analyses of health surveillance systems. Although these data might not be available to community members for ethical reasons around privacy or due to commercial restrictions by third parties, they can reveal the burden of vector-borne diseases and the need for community-serving research. As in the earlier example from Bunge and Warren, the transfer of knowledge regarding both vector-borne diseases and associated health indicators could lead to the identification of other health and wellbeing issues that are important to the local communities where the research is performed and that are not captured by health surveillance systems.

## Understand the local context of vector-borne disease transmission

Several disease-related factors are dependent on the local environment where transmission occurs; these include the emergence of vector-borne diseases, the likelihood that technologies can be generalised to control insect vectors elsewhere, and the potential to implement interventions at differ entspatial and temporal scales.^28^ As recognised in geography, ^29^ any environment exists beyond its place and location (ie, it is more than a simple container of living organisms and abiotic components) to include near and far dynamic relations among organisms and the abiotic resources that transform, define, and adapt to the environment. These relational environments, referred to as contexts in disease transmission,^30^ are of major importance for understanding constraints in vector-borne pathogen transmission and vector control efforts to reduce transmission. Although vector control can be central to reducing transmission, ^31^ in some contexts, vector control is not necessarily the best approach to reduce or eliminate transmission.

For example, malaria transmission in the WHO region of the Americas accounts for less than 1% of the global malaria burden.^3^ Unlike the WHO African region, where most malaria parasite transmission occurs, some middle-income countries in the Americas, such as Costa Rica, have developed health systems able to eliminate malaria through mass drug administration, the population-level delivery of curative treatments. ^32^ This system can more easily distribute drugs and ensure treatment adherence than control transmission by reducing mosquito populations, especially when primary vector species feed and rest outdoors. This is the case for *Anopheles albimanus*^33^ in Mesoamerica and the Caribbean, for which insecticide-treated nets, indoor residual spraying, and other control tools that work with mosquitoes that feed and rest inside homes are less effective. Even for indoor-resting *Anopheles gambiae* sensu stricto in sub-Saharan Africa,^31^ pressure from the use of long-lasting insecticidal nets and indoor residual spraying of insecticide can lead to shifts in parasite transmission ecology where new vector species, such as outdoor-biting *Anopheles arabiensis*, can reduce the effectiveness of these measures.^34^ Accordingly, the need to better understand the impacts of climate change on transmission in these situations is increasingly important. Trade-offs in transmission can also be particularly complex, with studies suggesting a competitive advantage for larvae of *An gambiae* sensu stricto co-occurring with *An arabiensis* as temperatures become warmer,^35^ highlighting the need for further field studies to understand malaria transmission ecology in sub-Saharan Africa in response to climate change.

Knowledge of the strengths and potential weaknesses of different local, national, and even regional health systems is key to understanding the feasibility of transmission reduction strategies in different contexts. This knowledge informs decisions regarding the translation of research and tools to a new setting, the feasibility of research plans in a given context, the quality of earlier observations made by community-affiliated and non-resident researchers, and the extent to which available data reveal previously ignored health problems. By contrast, an absence of contextual knowledge inherent to helicopter science (ie, the extractive practice of collecting or demanding local samples or data), coupled with the analysis of decontextualised data, ignorance of local research, and a failure to engage with local scientists and communities, prevents the cocreation of actionable knowledge,^36^ even preventing the control or elimination of local vector-borne diseases. For example, Panama has been very close to eliminating malaria for the last 15 years, but operational research activities coordinated by external groups favour external experts and consultants. The resulting operational research largely excludes local scientists and ignores previous local research, which shows that malaria transmission is concentrated in marginalised Indigenous populations. ^37^ Given that malaria severity and patterns of transmission are dependent on a complex array of underlying climate-dependent health and environmental factors, marginalised Indigenous populations, as suggested by local Panamanian scientists,^37^ would benefit from a wider scope of actions, beyond those seemingly most proximate to malaria—actions that are aimed at facilitating overall community health and wellbeing. Further, such community-serving operational research would enable a cocreation process, in which Indigenous knowledge and practices greatly enhance the successful translation to actionable research results.^37^ This point is well illustrated by contextualised research by scientific teams serving local populations to sustainably reduce domiciliary vector infestations to control Chagas disease (panel 1).

Localised ecological understanding is also key to learning more about vector-borne disease transmission. This principle becomes clear when evaluating the complex impacts of invasive species in new environments, which often directly connects plant health with human and animal health. For example, water hyacinth (*Eichhornia crassipes*), a conspicuous species, which has been repeatedly identified by residents of the areas that it has invaded, has adversely impacted native aquatic plant, fish, and bird species that depend on these habitats and, as a result, food security and animal and human health in these areas.^38^ Management has focused on the destructive removal of this highly invasive plant, as otherwise, its consequences are predicted to alter vector snail–parasite relationships in ways that could transiently increase schistosomiasis, a devastating global neglected tropical disease.^39^ Various strategies have been proposed to advance such understanding with local populations. For example, the Australian Department of Energy, Environment, and Climate Action^40^ focuses on spreading knowledge about water hyacinth and its damaging effects to culturally and linguistically diverse communities via largely one-way outreach strategies. As discussed in the next section, these kinds of engagement transfer some knowledge, but there is room to make the translation of both results and research more effective and more engaged.

## Render knowledge actionable

By linking research questions and framing hypotheses with ongoing efforts to improve health and wellbeing in the populations in which vector-borne diseases occur, research-based knowledge can become actionable. Actionable research is solution-focused, solidly grounded in disciplinary knowledge, and has outcomes that can be willingly adopted locally^9^ or in the voluntary renegotiation of common practices,^41^ or that aid decision making by local policy makers.^12,42^ In the case of vector control, actionable knowledge has been expressed as both bottom-up (ie, community-based approaches, such as the elimination of larval habitats) and top-down (ie, government-mandated and vertically structured practices, such as the large-scale application of insecticides by vector control specialists).^43^ In the case of *Aedes aegypti*-borne viruses,^43^ top-down approaches are effective at addressing sanitary emergencies. Nevertheless, bottom-up strategies are more sustainable and provide long-term benefits for the wellbeing of local populations (panel 1).^44^

However, not all community involvement implies bottom-up scientific practice. In some cases, local populations collect data for scientists (typically a top-down model of knowledge generation) and receive processed and interpreted results summaries, with all other aspects of the scientific process controlled by researchers, as currently practised in citizen science.^27^ This strategy is the dominant vector-borne disease research practice involving local communities in the USA and much of the Global North.^45^ In contrast, participatory action science engages scientists and local community members as partners in the coproduction of knowledge to solve community problems.^27^ This practice follows the approach pioneered in Detroit during the late 1960s described earlier.^26^ In participatory action science, local populations and scientists contribute as equal partners, from the conception of research to the proposal of actions that can decrease the origins or consequences of vector-borne diseases in community.^27^ This model of actionable knowledge in Latin America has focused on improving housing quality to reduce the likelihood of infestations by kissing bugs and, therefore, the incidence and prevalence of Chagas disease and other housing-related morbidities (panel 1; figure 1).

## Ask what else beyond climate change is affecting vector populations and transmission

Contemporary climate change is not an autonomous force of nature, but has been linked to anthropogenic activity that can be measured as carbon footprints or greenhouse gas emissions.^46^ These emissions illustrate the relational nature of environments, as emissions that originate remotely affect environments well beyond any kind of geopolitical or natural boundary (eg, countries in the Global North are responsible for most climate change causing emissions, yet the worst impacts of climate change are occurring in Global South countries, such as island nations facing the risk of disappearance as the sea level rises).^7,46^ From our understanding of the ecological niche of a species, we can infer that the multidimensional space over which organisms exist changes in response to how organisms dynamically transform their environment, a process in which niches are in constant construction.^47^ These realisations can help us to craft research questions that test hypotheses and generate community-serving actionable knowledge.

In the face of failing interventions that no longer reduce transmission, as has become common for insecticides with the evolution of resistance,^31^ we can formulate, test, and improve new ways to reduce vector-borne disease transmission. Novel approaches are based on emerging technologies for insect pest control^31,48^ or for pathogen elimination.^49^ For example, applying ecological approaches, such as push-pull integrated pest management, which combines methods that reduce vector contact with methods that restrict the environments where vectors can thrive, can enhance the impact of any single method on transmission.^50^ Indeed, over the last four decades, greener alternatives to vector management have been successful as they more clearly recognise the environmental nature of vector-borne diseases. The implementation of environmentally friendly strategies can engage community members who understand the ecology of transmission, as in the case of Chagas disease in Latin America (panel 1), dengue in wealthier contexts of the Global North,^51^ and mosquito-borne diseases more generally.^52^

Using the lens of environmental justice to frame the impacts of climate change on vector-borne diseases highlights the fact that the largest densities of vectors tend to be clustered around socioeconomically disadvantaged populations,^53,54^ which are generally also the populations most affected by climate change.^7,8^ In this context, community-serving research should adopt both environmental and population-based focuses, considering the social, political, economic, and demographic factors associated, for various reasons, with increased health inequities and disease risk. Policy analyses indicate that such approaches can accelerate malaria elimination.^32^ For example, the recognition in Costa Rica that malaria is an occupational disease of banana and pineapple plantation workers was key to developing policies and public health practices for these workers, such as mass drug administration, to substantially reduce malaria transmission during the 21st century.^32^ To enhance community-serving research, we must also recognise that communities are not reducible to demographic subsets of individuals. Communities have collective life and politics, complex debates, and differences, and often even structures of governance that need to be engaged and partnered with.^24^ For example, we have recognised that not engaging with the nuanced understandings and priorities of Indigenous communities is a major barrier for malaria elimination in Panama, where non-adherence to governmental malaria control policies is an act of resistance to an external government that is perceived as a threat to Indigenous identity and self-determination.^37^ By contrast, the understanding of communities beyond their demography, looking widely at their struggles and needs, has been key to the success of interventions that have reduced Chagas disease transmission across Latin America (panel 1).

In addition to an improved understanding of human–environment interactions in vector-borne diseases, community-serving research can help to define the poorly understood effects of extreme weather events and warming trends on shifts in vector species dominance and vectorial capacity. For example, following the 2011 tsunami in Japan, *Culex inatomii*, a nuisance marshland mosquito, became dominant in rice fields flooded with seawater.^55^ Although an extreme weather event did not trigger this shift, we could expect transient and permanent shifts in vector communities following climate change, particularly for vectors that can adapt readily to variable environments, such as *Aedes albopictus*^56^ and *Ae aegypti*.^57^ The issue of whether climate adaptation can drive the emergence of new vectors is also an important question. For example, physiological changes in common nuisance mosquitoes, such *Aedes sierrensis*,^58^ which is widely distributed across highlands in the western USA, could be associated with enhanced pathogen transmission competence and, conversely, adaptation could lead to an increased abundance of vectorially competent rare species, such as *Culex stigmatosa*, which can transmit West Nile virus.^59^ The latter phenomenon has been observed in Japan with *Aedes flavopictus*, a species competent for dengue virus transmission, which was previously rare but has become common in urban landscapes.^56,60^ When shifts in mosquito species also increase nuisance biting, local populations might perceive this change, creating an opportunity for participatory community-serving research. This research approach can benefit other questions in vector biology, for example, how vectorial competence changes in warm and variable environments.^61^ Such patterns could also be linked to land use and land cover transformations, which can drive stronger changes in transmission than warming trends.^62^ Land cover transformations and changes in use, and their impacts on changing everyday life, can also be perceived by local communities, as documented in rural communities engaged in agroecological food production practices.^63^

Ultimately, in developing community-serving science, asking what else beyond climate change affects vector populations and disease patterns in impacted areas can also become the basis for cocreating more nuanced research programmes. In such research programmes, research questions are crafted through community partnerships, with the dual aim of serving the community while testing hypotheses that advance understanding of the ecology and evolution of vectors and the diseases that they transmit.

## Ask how the scope of research-based knowledge can be broadened

As noted, community-serving research can improve population health and wellbeing beyond reducing or eliminating single vector-borne disease transmission. As illustrated by Chagas disease research in Latin America (panel 1), benefits from interventions aimed at reducing the transmission of one vector-borne disease can be robust enough to reduce the transmission of several infectious diseases.^42^ In this context, community-serving research can be applied with the view that diseases are not independent problems but interconnected expressions of common physiological processes at the individual level, connected with population-level social, economic, and environmental drivers that allow multiple infectious diseases to co-occur in the same vicinity. At the population level, frameworks that incorporate structural economic causes of disease persistence,^64^ commonalities among human, animal, and plant diseases,^48^ what we call the causes of the causes in syndemics,^65^ and the understanding of co-occurring diseases in complex ecosystems, where it is explicitly recognised that some populations live in disadvantaged socioeconomic and environmental conditions, are necessary for community-serving research.

In the context of vector-borne diseases, a broader relational perspective translates into integrated control methods for several vectors^66^ and the promotion of other approaches that can reduce transmission. As mentioned earlier (panel 1), improved housing quality can reduce the transmission of vector-borne, and other, diseases inside domiciles. Within the context of climate change, improved housing quality might also mitigate the health impacts of heatwaves, allergies, and environmental hazards overall.^7,67^ But when we ask why quality housing is not affordable to everyone, we are confronted by structural problems that shape disease and associated social and economic inequities. For example, political economies can implement policies, such as scaling-up production of a commodity or externalising (transferring to others) environmental and social costs while realising monetary profits for the few—actions that together can promote disease, even without changing the biology of pathogens and vectors.^64,67^ Broadening the scope of research beyond the silos that are barriers to knowledge and partnership is necessary for community-serving research, cocreating solutions to health problems, and improved wellbeing for everyone.

## Support communities to keep (or take back) what is theirs

**Figure 2:**
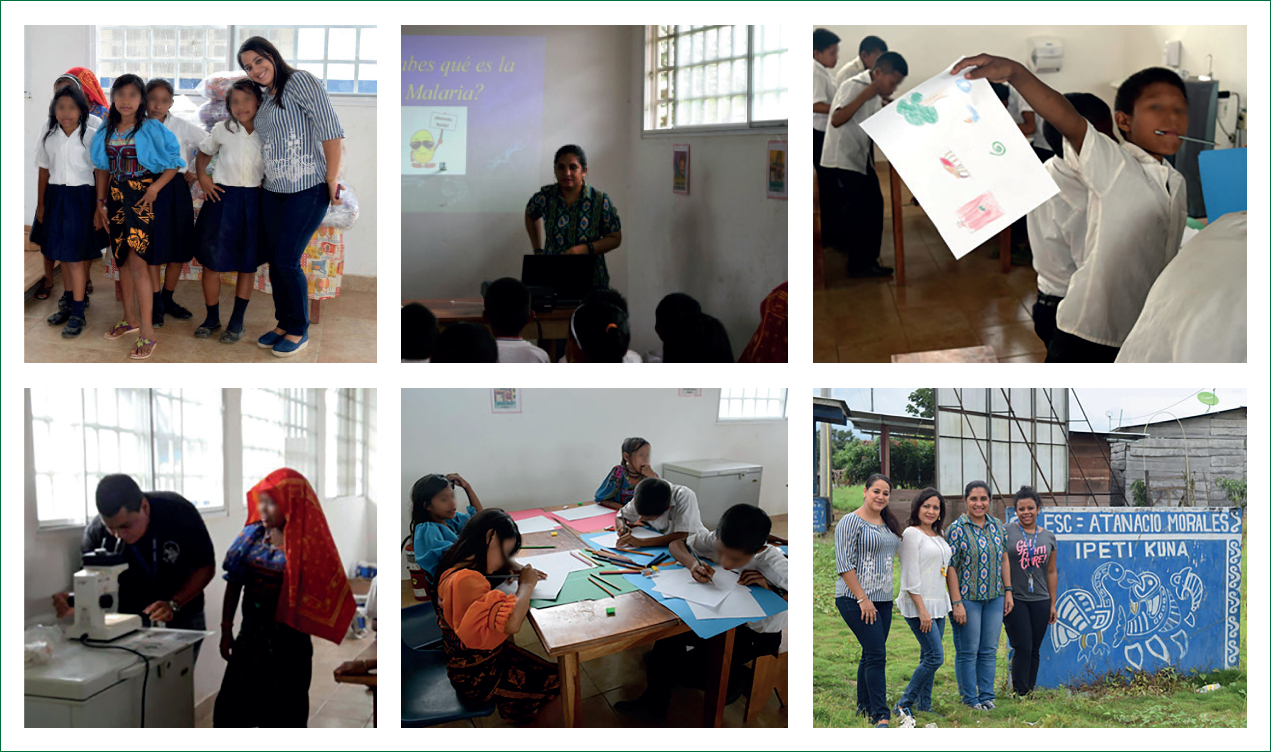
Community-serving research about malaria elimination under climate change Investigations to accelerate malaria elimination in Panama have been enabled by the coproduction and translation of knowledge by several tenure-track early-career scientists from the Gorgas Memorial Institute and Native Guna communities. Images depict different elements of knowledge cocreation where schoolchildren from Ipeti-Guna, an indigenous Guna community from Comarca Madugandi in western Panama, engaged in a hands-on learning experience about the transmission of *Plasmodium* parasites, the causative agents of malaria, by *Anopheles* mosquitoes and efforts in their community to support malaria elimination.^68^ This partnership has supported a local community to take back their knowledge. The next stage of malaria elimination in Panama can benefit from a more integrated knowledge cocreation process.

We propose that implementing mechanisms for the appropriation of research-generated knowledge by the people affected by vector-borne diseases is not zero-sum, but rather is additive, perhaps even non-linearly synergistic, with existing research strategies. In Panama, as part of our research on malaria elimination,^37^ we often discuss the ecology of transmission and our research findings with local populations (figure 2). Although we are not at the point of fully engaging in the cocreation process of participatory action science, we are working towards a more collaborative science where local communities influence the crafting of research questions and, when interested, are welcome to participate in aspects of data collection. The actionability of the knowledge generated by research will increase while improving practices and establishing truly integrated cocreated partnerships with local communities. This process is happening in community-serving research on Chagas disease throughout Latin America, but especially in Guatemala, as illustrated by efforts cocreated by Maria Carlota Monroy, other researchers at the Universidad de San Carlos de Guatemala, and several rural communities (panel 1). For incipient community-serving research practices, broadening training targets within the vector-borne disease and climate research communities can drive actionable knowledge and accelerate successful improvements in community health.

## Training everyone for community-serving research: from now and into the future

To achieve transmission reduction and elimination goals, we believe that research and training in vector biology must more commonly coproduce actionable knowledge on vectors and vector-borne disease transmission under climate change. Formal training programmes for participatory methods are expanding in both the Global South and the Global North (panel 2). We should also highlight the existence of comprehensive literature and guides on methodologies for participatory research.^69^ However, for many students, researchers, policy makers, and activists, the embodiment and detailed application of participatory methods are not intuitive, so acquiring knowledge and skills in these methods is crucial. In vector biology, beyond the case study of Chagas disease in Latin America (panel 1), we are aware of other successful examples illustrating actionable knowledge from community-serving research in vector-borne diseases.^51,52^ Vector biology is ready to follow the example of participatory cartography, practices whose diverse present-day relatives are still vibrant 60 years after Bunge, Warren, and others were doing their early work.^23–25^ Participatory cartography and geographical information science are even used for disaster risk reduction^70^ and, more broadly, participatory research has successfully rendered actionable knowledge from agroecology, food sovereignty, and food systems research.^71^

The acceleration of improved health outcomes depends on community-serving research and the engagement of research specialists whose expertise is allied to vector biology and disease ecology. We believe that climate, Earth, and atmospheric scientists can also engage in coproduction. For example, the European Space Agency, the Japan Aerospace Exploration Agency, the Japan Meteorological Agency, and the US-sponsored National Oceanic and Atmospheric Administration and National Aeronautics and Space Administration (NASA) have greatly increased public access of their Earth observations, including climate change data, through various open-source initiative programmes (panel 2).^72^ This access brings accountability to the work of these agencies and supports affiliated missions to promote transdisciplinary research.^73^ Community service remains aspirational at best for most agencies. Still, the Japan Meteorological Agency has advanced community service by triggering action plans when extreme weather events are forecasted to occur and affect local populations in Japan (panel 2). Through both community-serving research and community service, engaging members from wide segments of society at multiple levels is likely to reduce climate change denialism, an obstacle to progress linked to poor access to information and a paucity of positive engagement.^74^ Indeed, participatory roles are key to the most effective learning strategies, developing pedagogies where the learner is treated as a knowledge cocreator trained through dialogue and mutually shared experiences to liberate people from narrowly defined societal roles.^75^ Through this dialogue and efforts to communicate that research about climate change and its wider societal impacts, including those on health,^8^ are motivated by a desire to serve communities, we hope that this perspective helps to stimulate research practices that clearly articulate research as community-serving.

## Panel 1: Chagas disease research in Latin America

The history of many major vector-borne diseases tends to be associated with military conquest, colonialism, and imperialist expansion, a fact well illustrated by malaria and yellow fever.^10,11^ However, this was not the case for the description of American trypanosomiasis by Carlos Chagas in 1909, as he contributed these observations while providing health care to local underserved populations as part of an antimalarial campaign.^12^ As recounted in his description of *Trypanosoma cruzi*,^13^ the parasite causing Chagas disease, the disease was suspected to be a major cause of child mortality in the north of Minas Gerais state, Brazil. Chagas disease was also associated with a high prevalence of paediatric developmental and cognitive issues and heavy domiciliary infestations of kissing bugs.^13^ Descriptions of the disease in both Venezuela and Argentina were similar, as illustrated, respectively, by José Francisco Torrealba^14^ and Salvador Mazza,^15^ who described the disease as an expression of socioeconomic inequities. The history of Chagas disease has been uniquely marked by a fluid knowledge transfer with local populations, where clinical signs, such as the Romaña sign (figure 1A),^16^ earthen floors,^17^ and the invasion of homes by kissing bugs (figure 1B), have been recognised by rural populations throughout Latin America as indicators of *T cruzi* transmission and infection risk. As Chagas disease affects the poorest people in Latin America, multiple interventions have targeted transmission. In Costa Rica, efforts have included the removal of waste materials and woodpiles from peridomiciliary environments.^17^ Similarly, artificial habitats, such as dog kennels and chicken coops, can be managed to reduce human household colonisation by kissing bugs.^18^ In Guatemala, efforts focused on improving rural housing, including the use of wall plastering with locally available materials, and the implementation of ecological interventions, such as managing kissing bug refuges and alternative hosts to reduce housing infestation and transmission in the long term.^19^ The approach in Guatemala is an excellent example of knowledge coproduction as local populations were involved in aspects of the research and implementation, working alongside scientists in the process of housing improvement and environmental management. The impacts of these efforts extended well beyond the original goal of reducing the likelihood of kissing bug household reinfestation.^19^ In Venezuela, with the emergence of oral transmission of *T cruzi*, an emphasis has been placed on improving food management, especially with the preparation of fruit and sugarcane juices that do not enable the survival of *T cruzi*.^20^ In all of these efforts, it has become evident that success is closely tied with community participation in the research, an essential part of knowledge co-development, which extends beyond simply informing a community about research results.^21^

## Panel 2: Resources to develop participatory and community-serving research in the context of climate change

### Training in participatory methods:

- Ciencia Participativa is a network of programmes for training in participatory methods for Spanish and Portuguese-speaking audiences
- The Institute for Development Studies at the University of Sussex provides both online and in-person training and workshops in participatory methods
- ActionAid International offers the NetworkedToolbox with field-ready methods and tools for participatory methods
- The Public Science Project offers a summer Critical Participatory Action Research Institute in theory, ethics, and application of methods, which for the 2023 institute was targeted at graduate students, faculty, and members of community-based organisations

### Public climate, weather, and Earth observation data:

- The European Space Agency provides a mixed portal allowing climatic data access and describing the evidence for climate change
- The Japan Aerospace Exploration Agency portal on earth observations, including information about the use of satellite imagery to study climate change and natural disasters
- The Japan Meteorological Agency portal offers real-time information about weather, extreme climatic events, and disasters, and also provides information for action under dangerous environmental situations
- The National Oceanic and Atmospheric Administration has an open dissemination portal providing near real-time weather data
- NASA portal for science divulgation, including scientific data and some citizen science applications
